# Clinical nutrition care challenges in low-resource settings during the COVID-19 pandemic: A focus on Malawi

**DOI:** 10.7189/jogh.10.020363

**Published:** 2020-12

**Authors:** Bernadette Chimera, Isabel Potani, Allison I Daniel, Humphrey Chatenga

**Affiliations:** 1 School of Public Health and Family Medicine, University of Malawi, College of Medicine, Blantyre, Malawi; 2 Department of Nutritional Sciences, University of Toronto, Toronto, Canada; 3 Centre for Global Child Health, Hospital for Sick Children, Toronto, Canada; 4 The Childhood Acute Illness & Nutrition Network (CHAIN), Blantyre, Malawi; 5 Nutrition Group, North West University South Africa, Potchefstroom, South Africa

The coronavirus disease 2019 (COVID-19) pandemic has rapidly evolved and imposed extraordinary challenges to health care systems worldwide. Health systems in high-income countries have been overwhelmed due to severe COVID-19 infections, even though it causes serious illness in a relatively small fraction of patients who contract the virus. Therefore, it is expected that fragile health systems in African countries like Malawi will be highly vulnerable to the anticipated surge in severely ill patients with COVID-19.

Management of severe COVID-19 infection entails intensive monitoring and use of specialized medical equipment by clinical professionals including those with nutrition expertise like dietitians. To date, one of the major challenges faced in the implementation of public health measures in African countries has been inadequate nutrition for citizens living under the poverty line. In Malawi, clinical nutrition care is a crucial yet underdeveloped component of health care, hence, it is important to anticipate challenges exerted by COVID-19 on delivery of nutrition services.

Malawi, a landlocked low-income country with a population of almost 19 million, registered its first case on April 2, 2020 and had 3709 confirmed cases including 103 deaths as of July28, 2020. Like other countries globally, the COVID-19 response in Malawi has consisted of a myriad of interdisciplinary approaches aimed at case prevention and case management. The absence of a specific treatment for this novel virus has called for pragmatic prevention and management strategies based on clinical and public health experience. The preventive approaches aimed to reduce transmission rate include promotion of handwashing and physical distancing also in form of lockdowns. However, the appropriateness, effectiveness, feasibility, and sustainability of these measures are unknown and questionable particularly in low-resource settings like Malawi, the only country to announce a lockdown and cancel it before its start. In this paper we discuss the anticipated challenges in providing adequate nutrition and preventing malnutrition-related morbidity and deaths in the public health system in Malawi.

## NUTRITION AND COVID-19 INFECTION

Inadequate nutrition remains a global health problem, having the highest prevalence and impact in low- and middle-income countries (LMICs). The bidirectional relationship between nutritional status and immunity poses a likely threat of COVID-19 pandemic exacerbating the burden of global malnutrition.

Nutritional status is expected to be a highly important factor related to outcomes of patients with COVID-19. It has been documented that major causes of morbidity and mortality in COVID-19 patients are secondary to complications of acute respiratory syndrome related to prolonged Intensive Care Unit (ICU) stay [1]. ICU stays, and particularly their longer duration, are well-documented causes of malnutrition, with loss of skeletal muscle mass and function which in turn may lead to poor quality of life, disability, and morbidity long after ICU discharge [2].

Non-communicable diseases (NCDs) like obesity, diabetes, and hypertension are also now known risk factors for poor clinical outcomes in people hospitalized with COVID-19 [3]. With an increasing prevalence of obesity and metabolic diseases in Malawi, these risk factors could increase risk of morbidity and mortality associated with COVID-19. On the other end of the spectrum, age above 65 years, another COVID-19 risk factor, coexists with markers of poor nutrition status such as sarcopenia irrespective of body mass index [4]. Due to the younger demographic in Malawi, old age may not be a significant risk factor compared to high-income settings. However, immunosuppression and malnutrition secondary to infectious diseases such HIV and tuberculosis may be associated with greater risk, though data on the relationship between COVID-19 outcomes and these risk factors are yet to be available.

## CHALLENGES IN CLINICAL NUTRITION CARE

### Inpatient care

To achieve COVID-19 nutrition recommendations outlined by various authorities in nutrition practice, countries must already have adequate human resource capacity in place. In settings such as Malawi where there are only 11 clinical dietitians in the country, a lack of human resource in clinical nutrition provision is anticipated to rank high on the list of challenges.

A study by Bunyani et al [5] in Lilongwe, Malawi, indicated numerous challenges in hospital nutrition care delivery, including a lack of a nutrition department with qualified nutritionists or dietitians in their respective hospitals and absence of nutrition protocols for acutely critically ill adult patients in the wards. This therefore poses a challenge in terms of having the capacity to conduct nutrition screening and assessment of COVID-19 patients, particularly in ICUs, in the absence of nutrition protocols and personnel. In 2018, the Malawi government introduced two dietitians in tertiary level health facilities, yet the patient to dietitian ratio remains too large to cater for the population in need. It is also notable that qualitative research around a nutrition support program led by a dietitian at Queen Elizabeth Central Hospital in Blantyre showed that nurses and physicians who worked alongside the dietitian valued the role of nutrition support in improving quality of care [6].

**Figure:**
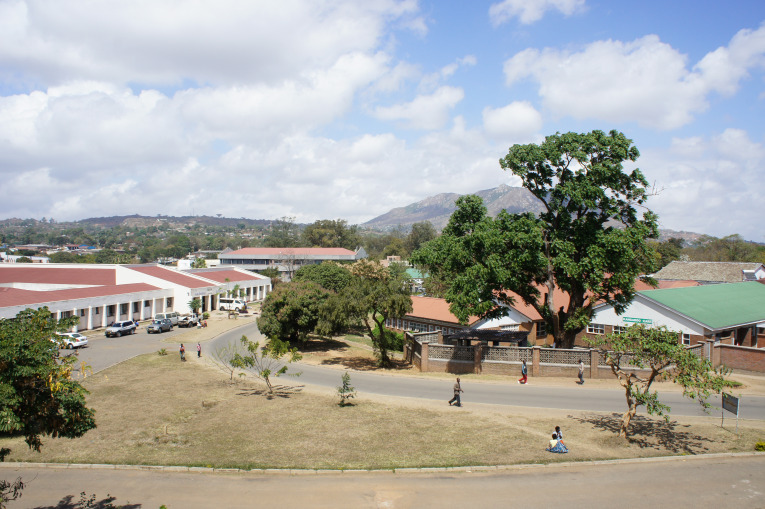
Photo: Queen Elizabeth Central Hospital, Blantyre, Malawi. One of the largest tertiary hospitals in Malawi, equipped with a COVID-19 isolation centre.

In the absence of a COVID-19-specific clinical nutrition response, hospital malnutrition will likely increase in both COVID-19 and other patients in low-income settings like Malawi. Literature shows [7] that 20% of COVID-19 cases require ventilatory support for approximately 10-14 days, therefore demonstrating the need for specialized nutrition support such as enteral and parenteral feeding. However, due to the lack of capacity in hospital pharmacies in Malawi to prepare or procure standard nutritional supplements and feeds for patients, these types of feeding cannot be easily achieved. This will increase the risk of malnutrition for all patients anticipated to be on ventilator support for a prolonged duration.

In addition to this, the feeding of patients within hospitals in Malawi largely depends on food prepared and served by a patient’s designated guardian, to supplement inadequate hospital meals. Guardians are typically allowed to visit a hospitalized patient three times per day. However, due to the World Health Organization recommendation of physical distancing, visits have been banned at hospitals. These restrictions will negatively affect the availability of food for patients, resulting in an increased risk in malnutrition amongst hospitalized patients with either COVID-19 or other conditions as a result of inadequate intake.

Aside from the effects of COVID-19 on the adult population, we forecast the presence of indirect negative effects on child health and nutrition. This could be attributed to numerous factors which may include ill caregivers failing to provide adequate nutrition through breastfeeding (though it is currently recommended to continue breastfeeding even in mothers with COVID-19), health care failures associated with movement restrictions, economic disruptions, and limited food access. Further to this, efforts made by already fragile health systems to prepare for COVID-19 at the possible expense of other health services, which may include food security and nutrition programs, may also contribute to the rise in severe acute malnutrition admissions.

### Outpatient care

Due to the novelty of the COVID-19 pandemic, many assumptions about its potential effects on other health services have been extrapolated from other contexts. For example, the Ebola outbreak in West Africa caused a health care shutdown, which resulted in outpatient care coverage declining by approximately 27% [8]. In India, during the current pandemic, a strict lockdown resulted in a 40%-50% decrease in outpatient NCDs services [9]. Currently in Malawi, NCD outpatient care has been scaled down in diabetes and hypertension outpatient clinics by only seeing newly diagnosed patients. This means services such as face-to-face individualized nutrition counselling provided by a dietitian is only reserved for new diagnosis patients and therefore there is no follow-up for compliance to dietary advice and control of blood glucose and blood pressure levels after this initial visit. Poor NCD management may result in an increase in incidence, severity, and potentially mortality due to NCDs.

Physical distancing, which is currently promoted in Malawi, could inadvertently cause a rise in poor management of NCDs from a lack of access to nutritious foods combined with low physical activity. Furthermore, income losses related to market diversity restrictions, movement restrictions may also contribute to a decline in diet quality, especially as more nutritious foods are often costlier than energy-dense foods.

## RECOMMENDATIONS FOR CLINICAL NUTRITION IN MALAWI

To address misinformation, related to how COVID-19 is transmitted and treated, advocacy and risk communication through health literacy campaigns should also include nutrition education. As shown by evidence from the WHO [10], campaigns around health literacy can help in reducing long-term effects of COVID-19 on nutrition.

Deriving from lessons learned from the Ebola crisis in West Africa, we also agree that the public health response should aim to maintain utilization of routine health services [8]. We commend the Malawi government for initiating recruitment of frontline health workers such as doctors and health surveillance officers during the pandemic. However, there is need to include nutrition personnel such as dietitians in the COVID-19 response at the facility level in order to address both immediate and enduring nutrition challenges posed by COVID-19.

To complement the recruitment of nutrition personnel, we propose the initiation of training for nutrition management in pandemics for all frontline workers especially in a setting such as Malawi where clinical nutrition specialists are scarce. Moving forward, LMICs such as Malawi also need to invest in inclusion of standard enteral and parenteral feeding products in the essential medicines list, which become particularly important for severe infections such as COVID-19 and future outbreaks.
